# Detection of Tn*7*-Like Transposons and Antibiotic Resistance in *Enterobacterales* From Animals Used for Food Production With Identification of Three Novel Transposons Tn*6813*, Tn*6814*, and Tn*6765*

**DOI:** 10.3389/fmicb.2020.02049

**Published:** 2020-09-04

**Authors:** Juan He, Cui Li, Pengfei Cui, Hongning Wang

**Affiliations:** Animal Disease Prevention and Food Safety Key Laboratory of Sichuan Province, Key Laboratory of Bio-Resource and Eco-Environment of Ministry of Education, College of Life Sciences, Sichuan University, Chengdu, China

**Keywords:** Tn*7*-like transposons, *Enterobacterales*, antibiotic resistance, class 2 integrons, animals used for food production

## Abstract

*Enterobacterales* are widely distributed in the gastro-intestinal system of animals and may cause opportunistic infections. Worse still, multidrug-resistant *Enterobacterales* also poses a serious threat to public health. Tn*7*-like transposons have been found in several species of the *Enterobacterales* order and play an important role in dissemination of antibiotic resistance. This study aimed to investigate the distribution and genetic characterization of Tn*7*-like transposons in *Enterobacterales* isolates from food animals and their association with antibiotic resistance. *Enterobacterales* isolated from the samples were identified and classified according to the 16S rDNA sequence. Tn*7*-like transposons and associated integrons were detected by polymerase chain reaction (PCR) and sequencing. The antibiotic resistance of each Tn*7*-like transposon positive isolate was detected according to the Kirby-Bauer disk diffusion method. Then, six representative strains were selected to study the genetic environment by whole-genome sequencing (WGS). In total, we isolated 377 Tn*7*-like transposons positive strains of *Enterobacterales*. Class 2 integrons were detected in 99.5% of the isolates, and there were high frequency mutation sites especially in base 535, a stop mutation. Many isolates (54.9%) were multidrug-resistant and observed high resistance rates to trimethoprim/sulfamethoxazole and streptomycin. Among these strains, we found three new types of Tn*7*-like transposons, named Tn*6813*, Tn*6814*, and Tn*6765*. This is the first comprehensive survey that shows Tn*7*-like transposons in *Enterobacterales* from animals used for food production in different regions of China. This study also provides an insight into the horizontal transfer of resistance genes associated with Tn*7*-like transposons.

## Introduction

Several genera and species among the *Enterobacterales* are widely distributed in humans and other animals (Sassone-Corsi et al., 2016). The *Enterobacterales* order includes *Escherichia*, *Salmonella*, *Klebsiella*, *Proteus*, *Morganella*, and *Providencia* among other genera. Although *Enterobacterales* often comprise less than 1% of a healthy intestine’s microbiota, some *Enterobacterales* are also encountered in the inflamed gut and cause urinary tract infections, septicaemia, pneumonia, and other intra-abdominal infections (Lupo et al., 2013; Toombs-Ruane et al., 2017). To prevent and treat these infections caused by *Enterobacterales*, many antibiotics are usually used in animals. However, nonsystematic use or misuse of antibiotics have resulted in the emergence of antibiotic-resistant variants and a corresponding increase in the failure rate of these agents for treating bacterial diseases (Marshall and Levy, 2011).

Antimicrobial resistance (AMR) in *Enterobacterales* has emerged as a problem in both human and veterinary medicine (Livermore, 2009; von Tippelskirch et al., 2018), and the interest of the scientific community in the presence and circulation of resistant organisms from animals used for food production has also increased due to the important public health implications (Meunier et al., 2006; Cortes et al., 2010; Goncalves et al., 2010).

A common form of resistance dissemination in *Enterobacterales* is mediated by transposons. Tn*7*-like transposons, as the Tn*7* transposon derivatives, have been found in several species of the *Enterobacterales* order, such as *Proteus mirabilis* and *Morganella morganii* (Chen et al., 2018, 2019). This kind of transposons carries a great diversity of antimicrobial resistance genes (ARGs).

Highly conserved, the sequences of the two ends of the Tn*7* transposon are encoding transposition module and class 2 integron system. The transposition module encodes five proteins required for two transposition pathways, TnsA, TnsB, TnsC, TnsD, and TnsE (TnsABCDE; Peters and Craig, 2001b). The class 2 integron of Tn*7* transposon has an organization similar to that of the class 1 integron and carries three resistance gene cassettes—*aadA1*, *sat*, and *dfrA1* (Sundstrom et al., 1988, 1991; Sundstrom and Skold, 1990)—close to an open reading frame (ORF), *intI2*. *intI2* has premature translation termination due to mutation of base 535 encoding integrase from C to T, thus with no function. Although these gene cassettes are fixed in Tn*7* transposons due to mutations in the homologous recombinase, they can be rearranged in hosts expressing the relevant recombinase, resulting in other combinations of antibiotic resistance genes (Hansson et al., 2002).

The Tn*7*-like transposons, as important mobile platforms to transfer bacterial AMR, transfer various resistance genes among bacteria through their transposase, promoting the horizontal spread of drug resistance in bacteria. However, there is a paucity of data on the comprehensive analysis of Tn*7*-like transposons in antibiotic resistant *Enterobacterales* isolates. In this study, we have determined the incidence of Tn*7*-like transposons and their associated integrons and dealt with detailed genetic characterization of three novel transposons with complex mosaic structures in a collection of random *Enterobacterales* isolates collected from animals used for food production in China. Additionally, the possible association between the occurrence of Tn*7*-like transposons and antibiotic resistance phenotypes was also determined by statistical analysis.

## Materials and Methods

### Isolation and Identification of *Enterobacterales*

A total of 1,474 consecutive and unduplicated clinical isolates of *Enterobacterales* were collected from animals used for food production on 10 poultry and 12 swine farms in eight provinces in China between June 2018 and January 2020 (Table 1). All the isolates were presumptively identified through phenotypic methods, including colony morphology on MacConkey Agar (Land Bridge, Beijing, China) or Eosin-Methylene Blue Agar (Land Bridge, Beijing, China). The identification of these isolates was later confirmed using 16S rDNA gene sequencing. All *Enterobacterales* strains were stored at −20°C in 25% glycerol.

**Table 1:** Bacterial strains isolated from 2018 to 2020.

Source	Species	No. of strains	Note
Pigs	*Escherichia coli*	413	Fecal sample, cloacal swab, drinking water, or small intestine of swines from 12 swine farms in six different provinces of China.
	*Proteus* spp.	208	
	*Providencia* spp.	164	
	*Morganella morganii*	146	
	*Klebsiella pneumoniae*	141	
	*Salmonella enterica*	82	
Total		1,154	
Chicken	*Escherichia coli*	121	Fecal sample, cloacal swab, drinking water, or small intestine of chicken from 10 poultry farms in five different provinces of China.
	*Proteus* spp.	53	
	*Providencia* spp.	46	
	*Klebsiella pneumoniae*	24	
	*Salmonella enterica*	76	
Total		320	

The animal study was reviewed and approved by the College of Life Science, Sichuan University affiliation ethics committee, and all efforts were made to minimize animal suffering.

### Detection of Tn*7*-Like Transposons by Polymerase Chain Reaction

All 1,474 *Enterobacterales* isolates in this study were screened for the presence of Tn*7*-like transposons using a PCR-based method targeting *tnsA*, *tnsB*, and *tnsC* genes, which encode three conserved Tn*7* transposases (Peters and Craig, 2001b; Peters, 2014). The isolates were grown overnight (18–24 h) in Brain Heart Infusion (BHI) broth (Oxoid, Basingstoke, UK) at 37°C with a rotation speed of 200 rpm, and the DNA template was prepared using the boiling method (Lévesque et al., 1995). The PCR mixture was prepared with a final volume of 20 μl, containing 1 μl of template DNA, 8 μl ddH_2_O, 10 μl Taq PCR MasterMix, and 0.5 μl each primer. The specific primers for detecting *tnsA*, *tnsB*, and *tnsC* genes are shown in Supplementary Table S1. Positive PCR products were sequenced by Chengdu Sangon Biological Engineering Technology & Services Co, Ltd.

### Phenotypic Evaluation of Antibiotic Resistance

The antibiotic resistance profile of all Tn*7*-like transposons positive isolates were determined according to the Clinical and Laboratory Standards Institute (Clinical and Laboratory Standards Institute [CLSI], 2017) guidelines. The following antimicrobials (all discs from Oxoid, Basingstoke, UK) were used: amikacin (AMK, 30 μg), imipenem (IPM, 10 μg), cefoxitin (FOX, 30 μg), ciprofloxacin (CIP, 5 μg), streptomycin (STR, 10 μg), gentamicin (GEN, 10 μg), florfenicol (FLR, 30 μg), aztreonam (ATM, 30 μg), ceftazidime (CAZ, 30 μg), and trimethoprim/sulfamethoxazole (SXT, 1.25/23.75 μg). *Escherichia coli* ATCC 25922 was used as a quality control strain.

### Incidence of the Integrons and Associated ARGs

The isolated Tn*7*-like transposons positive *Enterobacterales* strains were checked for the presence of *intl2* integrase genes by PCR, using primers (Supplementary Table S1) and methodology described previously (Cocchi et al., 2007; Rehman et al., 2017). These mutation site sequences of *intI2* were visualized with Weblogo^1^.

### WGS and Analysis

We also analyzed genetic environment of Tn*7*-like transposons among strains exhibiting unique resistance phenotypes. The whole genome of the Tn*7*-like transposons positive strain was sequenced using Illumina MiSeq with a 200-fold sequencing depth and Nanopore PromethION platform with a 100-fold sequencing depth (Novogene Technology Co., Beijing, China). Genome assembly was carried out by *de novo* assembly with Unicycler v0.4.7, and the sequence was annotated using the NCBI Prokaryotic Genome Annotation Pipeline v4.2. Mobile elements, resistance genes, and other features were annotated by INTEGRALL (Moura et al., 2009), ISfinder (Siguier et al., 2006), ResFinder (Kleinheinz et al., 2014), PlasmidFinder (Carattoli et al., 2014), and the Tn Number Registry (Roberts et al., 2008) online databases, and the analysis was conducted using the BLAST program^2^.

### Horizontal Transfer and Stability of Tn*7*-Like Transposons

Conjugation was performed using rifampin-resistant *E. coli* EC600 as the recipient and the *P. mirabilis* SCBX1.1 isolate as the donor with selection on *Salmonella Shigella* agar (Land Bridge, Beijing, China) plates containing 300 μg/ml rifampicin and 10.24 μg/ml flufenicol. Successful horizontal transfer of plasmid p1.1.2 containing Tn*7*-like transposon was confirmed using antibiotic sensitivity test and PCR, and then, the conjugation frequency was calculated as transconjugants divided by number of donors (Dong et al., 2019). The stability of Tn*6765*, Tn*6813*, and Tn*6814* was determined by passage in BHI broth lacking antibiotics as it was described previously (Sandegren et al., 2012).

### Statistical Analysis

Variables are expressed as percentages (%). All statistical analyses were conducted with the GraphPad Prism 8 software. Chi square test for samples were used. Value of *p* < 0.05 was considered significant.

### GenBank Accession Numbers

The complete sequences of p1.1.1 (CP047113), p1.1.2 (CP047114), and all Tn*7*-like transposons, Tn*6763* (MN641830), Tn*6764* (MN628641), Tn*6817* (MT469878), Tn*6813* (MT469876), Tn*6814* (MT469877), and Tn*6765* (MT503200), identified in the present study were submitted to NCBI GenBank.

## Results

### Incidence of Tn*7*-Like Transposons in *Enterobacterales*

A total of 1,474 strains of *Enterobacterales* were isolated through analysis of samples collected from Sichuan, Hainan, Chongqing, Shandong, Hebei, Xizang, Liaoning, and Anhui provinces. Of the 1,474 *Enterobacterales* strains examined, 377 strains contained Tn*7*-like transposons. They included 128 (24.0%) *E. coli*, 150 (57.5%) *Proteus* spp., 48 (22.9%) *Providencia* spp., 21 (14.4%) *Morganella morganii*, 17 (10.3%) *Klebsiella pneumoniae*, and 13 (8.2%) *Salmonella enterica*. Statistical analysis showed that the prevalence of Tn*7*-like transposons was different among different bacteria genera (*p* < 0.0001; Chi square test). The Tn*7*-like transposons positive rate of *Proteus* spp. was significantly higher than other bacteria’s (*p* < 0.0001; Chi square test; Table 2).

**Table 2:** The separation rate of Tn*7*-like transposons in *Enterobacterales* of different genera.

Species	Total isolates	Tn*7*-like-positive isolates	Separation rate, %
*E. coli*	534	128	24.0^b^
*Proteus* spp.	261	150	57.5^a^
*Providencia* spp.	210	48	22.9^bc^
*M. morganii*	146	21	14.4^cd^
*K. pneumoniae*	165	17	10.3^d^
*S. enterica*	158	13	8.2^d^

Chi-square test was used to analyze the data. Data labeled with entirely different letters were significantly different (*p* < 0.05).

### Antimicrobial Resistance Phenotypes

Among the isolates, 207 (54.9%) were multi-drug resistant (MDR, resistant to at least three different classes of antibiotics). *Proteus* spp. and *S. enterica* had the higher multidrug resistance rates of 59.3 and 61.5%, respectively. Notably, high resistance rates were observed for streptomycin (87.8%) and trimethoprim/sulfamethoxazole (74.0%), followed by the rates for florfenicol (60.5%), gentamicin (24.4%), and ciprofloxacin (22%). Among them, resistance rates to gentamicin and ciprofloxacin were higher in *Proteus* spp. (30.7 and 28.0%) and *S. enterica* (38.5 and 23.1%). Low resistance rates to amikacin (6.1%), ceftazidime (5.8%), cefoxitin (5.8%), and aztreonam (4.2%) were detected, whereas *S. enterica* was more resistant than other *Enterobacterales* strains to ceftazidime and aztreonam (38.5 and 46.2%). Resistance to imipenem (1.6%) was only observed in *E. coli*, and most of the *Enterobacterales* isolates were highly susceptible to imipenem (Table 3). No significant difference was found between the antibiotic resistant profiles of isolates from pigs and chicken (Supplementary Table S2).

**Table 3:** Rates of resistance to antimicrobial agents of Tn*7*-like transposons positive isolates.

Antimicrobial agent	Breakpoints (mm)			% resistant isolates						
	*R*(≤)	I	*S*(≥)	*E. coli* (*n* = 128)	*Proteus* spp. (*n* = 150)	*Providencia* spp. (*n* = 48)	*M. morganii* (*n* = 21)	*K. pneumoniae* (*n* = 17)	*S. enterica* (*n* = 13)	Total (*n* = 377)
Gentamicin (GEN, 10 μg)	12	13–14	15	24.2	30.7	12.5	9.5	11.8	38.5	24.4
Streptomycin (STR, 10 μg)	11	12–14	15	84.4	90.0	95.8	90.5	70.6	84.6	87.8
Florfenicol (FLR, 30 μg)	14	15–18	19	60.9	59.3	56.3	76.2	64.7	53.9	60.5
Ceftazidime (CAZ, 30 μg)	17	18–20	21	6.3	3.3	8.3	0.0	0.0	38.5	5.8
Imipenem (IPM, 10 μg)	19	20–22	23	1.6	0.0	0.0	0.0	0.0	0.0	0.5
Cefoxitin (FOX, 30 μg)	14	15–17	18	7.0	6.0	2.1	0.0	11.8	7.7	5.8
Trimethoprim/sulfamethoxazole (SXT, 1.25/23.75 μg)	10	11–15	16	68.0	85.3	75.0	52.4	41.2	76.9	74.0
Aztreonam (ATM,30 μg)	17	18–20	21	3.9	1.3	4.2	0.0	5.9	46.2	4.2
Ciprofloxacin (CIP, 5 μg)	15	16–20	21	22.7	28.0	16.7	4.8	0.0	23.1	22.0
Amikacin (AMK, 30 μg)	14	15–16	17	6.3	7.3	4.2	4.8	5.9	0.0	6.1
Multi-drug resistant (MDR)				55.5	59.3	43.8	42.9	52.9	61.5	54.9

R, resistant; I, intermediate; S, susceptible.

### Distribution of Tn*7*-Like Transposons Associated Integrons

**Figure 1:**
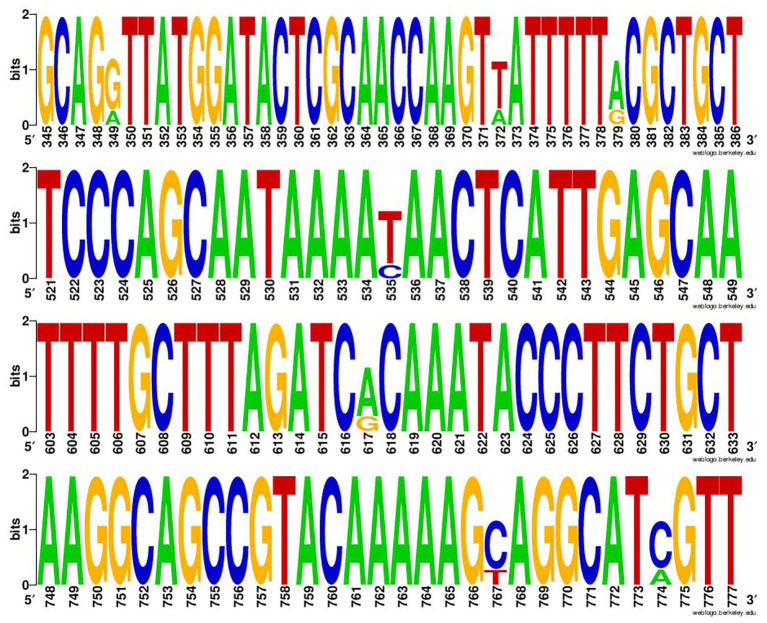
The Weblogo of repeats of *intI2*. The sequences were each mutation site of the integrase gene of class 2 integrons.

The positive rate of class 2 integrons in 377 *Enterobacterales* strains was 99.5% (two strains of *E. coli* lacked *intI2*). Through sequencing and sequence comparison, the results showed that *intI2* usually had mutation sites, and most of them were at position 349, 372, 379, 535, 617, 767, and 774, among which 535 base mutations (C mutated to T) were terminating mutations (Figure 1).

### Genetic Characterization of Tn*7*-Like Transposons

**Figure 2:**
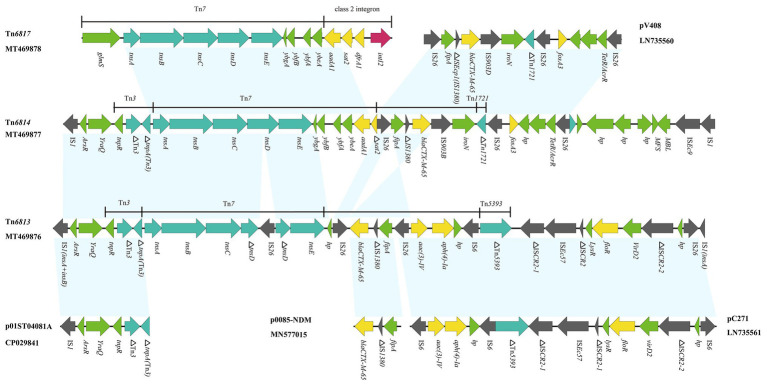
Genetic structure of Tn*6813*, Tn*6814*, and Tn*6817*. The physical maps were generated using Easyfig 2.2.3 and DNAMAN Version 8.0. Linear comparison of Tn*6813* region in *E. coli* strain SFE8 with Tn*6814* region in *E. coli* strain SCZE5, and Tn*6817* region in *S. enterica* strain SCFS4. Genes and open reading frames (ORFs) are shown as arrows, and their orientations of transcription are indicated by the arrowheads. Horizontal lines, different regions corresponding to Tn*7*, Tn*1721*, Tn*3*, Tn*5393*, and integrons. Antimicrobial resistance genes are in yellow, transposase are in blue, and integrase genes are in red. The IS elements are indicated by gray arrows. Other functions or putative proteins are in green. Shared regions with 99% identity are indicated by shading.

**Figure 3:**
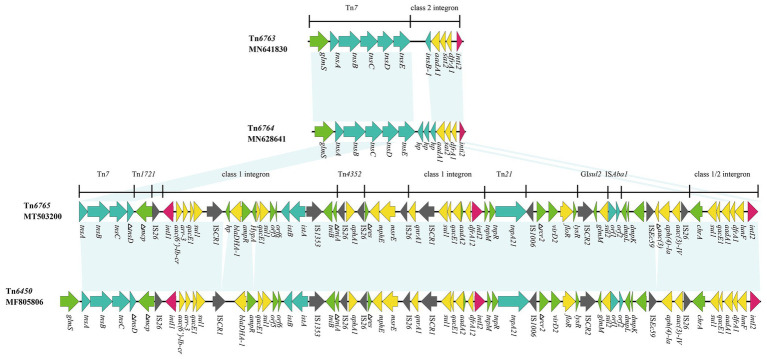
Genetic structure of Tn*6763*, Tn*6764*, and Tn*6765*. The physical maps were generated using Easyfig 2.2.3 and DNAMAN Version 8.0. Linear comparison of Tn*6765* region in p1.1.2 with Tn*6763* region in *P. mirabilis* strain, Tn*6764* region in *E. coli* strain and Tn*6450* region in *P. mirabilis* strain SNYG17 (GenBank accession number MF805806). Genes and ORFs are shown as arrows, and their orientations of transcription are indicated by the arrowheads. Horizontal lines, different regions corresponding to Tn*7*, Tn*1721*, Tn*21*, and integrons. Antimicrobial resistance genes are in yellow, transposase are in blue, and integrase genes are in red. The IS elements are indicated by gray arrows. Other functions or putative proteins are in green. Shared regions with 99% identity are indicated by shading.

Among the Tn*7*-like transposons carrying strains, six multi-drug resistant isolates were randomly selected for whole-genome sequencing, and yielded six completed Tn*7*-like transposons genetic structures. According to the transposon nomenclature^3^, we designated them Tn*6763*, Tn*6764*, Tn*6765*, Tn*6813*, Tn*6814*, and Tn*6817*. Tn*6763* (GenBank accession number MN641830), Tn*6764* (GenBank accession number MN628641), and Tn*6817* (GenBank accession number MT469878) were located on the chromosomes of *P. mirabilis*, *E. coli*, and *S. enterica*, respectively. They were typical Tn*7* transposons comprising the transposition module (*tnsA*, *tnsB*, *tnsC*, *tnsD*, and *tnsE*), three gene cassettes (*aadA1*, *sat2*, and *dfrA1*), and an inactive class 2 integrase gene (Figures 2, 3).

Tn*6813* (GenBank accession number MT469876) and Tn*6814* (GenBank accession number MT469877) were novel Tn*7*-like transposons in chromosomes, acquired from *E. coli* SFE8 and SCZE5, respectively. Tn*6813* was 32,688 bp in size and Tn*6814* was 32,874 bp. The structures of the two Tn*7*-like transposons were respectively surrounded by two IS*1* elements, while one IS*1* of Tn*6813* was incomplete with missing C-terminus. Transposition module (*tnsA*, *tnsB*, *tnsC*, *tnsD*, and *tnsE*) of Tn*7* in Tn*6814* was intact, but *tnsD* in Tn*6813* was truncated by an IS*26* element. Compared to canonical Tn*7*, the area’s downstream of transposition modules of Tn*6813* and Tn*6814* differed by deletions involving class 2 integrons and its associated gene cassettes. Actually, Tn*6813* had no *intI2* and no gene cassettes (*dfrA1*-*sat2*-*aadA1*), and Tn*6814* had only an *aadA1* and an incomplete *sat2* gene left (Figure 2).

Tn*6765* (GenBank accession number MT503200) was located on a plasmid of *P. mirabilis* SCBX1.1, named p1.1.2 (GenBank accession number CP047114). Sequence analysis showed that 19 resistance genes, except *cfr* and *erm(B)*, were carried by the Tn*7*-like transposon (Figure 3). The novel MDR transposon harbored different resistance genes, including *bla*_DHA-1_ (cephalosporin resistance), *qnrA1* (fluoroquinolone), *aac(6’)-Ib-cr* (fluoroquinolone and aminoglycosides), *floR* (chloramphenicol/florfenicol), *mphE* and *msrE* (macrolide), and *lunF* (lincosamide) genes. However, the *cfr* and *erm(B)* gene was carried by another 12,795 bp plasmid p1.1.1 (Supplementary Figure S1; GenBank accession number CP047113), which existed in the same strain as p1.1.2 (Figure 3).

Tn*6765* was 64,752 bp (corresponding to bases 47,309–112,060 in GenBank accession number CP047114) with an average GC content of 52.94% that differed from that of the rest of the *P. mirabilis* plasmid (GC content, 44.35%). It had partial characteristics of the Tn*7* transposon, which contained transposase genes *tnsA*, *tnsB*, and *tnsC*. But its transposition module lost *tnsE* gene and the *tnsD* was truncated by insertion of the inverted repeat (IR mcp) of Tn*1721* (Figure 3).

**Figure 4:**
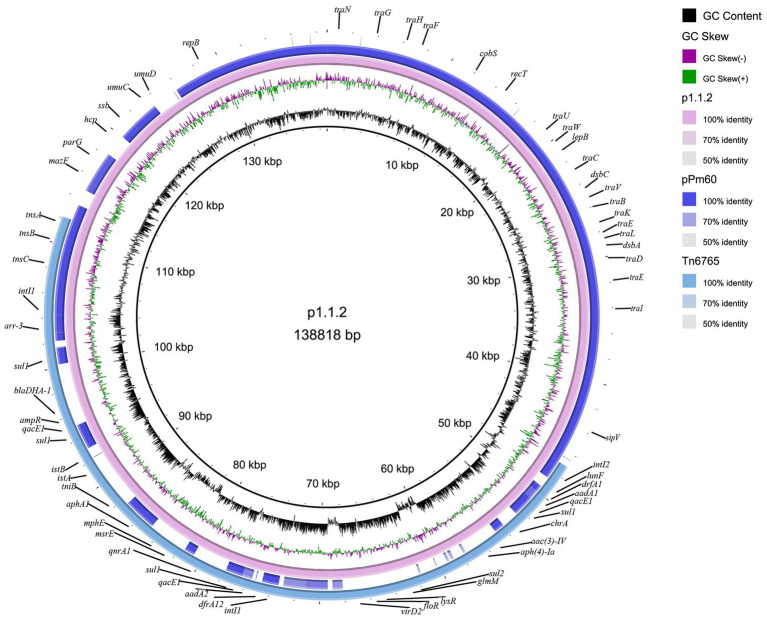
Circular representation of plasmid p1.1.2. The physical map of p1.1.2 was generated using BRIG v0.95.

The plasmid carrying Tn*6765*, named as p1.1.2, was 138,818 bp in size and had a GC content of 44.35%. Blastn results showed that, except the Tn*6765* region, other regions of plasmid p1.1.2. showed 99.98% similarity and 91% coverage with nucleic acid sequence of *P. mirabilis* plasmid pPm60 (GenBank accession number MG516911), which also carried the Tn*7*-like transposon published in NCBI database (Figure 4).

### Horizontal Transfer and Stability of Tn*7*-Like Transposons

Double-antibiotics Salmonella Shigella agar plates (300 μg/ml rifampicin + 10.24 μg/ml flufenicol) were used to screen the transconjugant. The results of drug sensitivity, electrophoresis, and sequencing showed that the plasmid p1.1.2 could be successfully transferred to *E. coli* EC600 (Supplementary Table S3). A novel Tn*7*-like transposon (Tn*6765*) transconjugant was obtained through the conjugative transfer test and at a frequency of 1.5 × 10^−4^ transconjugants per donor (average of three independent determinations). The stability of Tn*6765*, Tn*6813*, and Tn*6814* was determined by picking 100 clones in the twenty first passage to detect the presence of Tn*6765*, Tn*6813*, and Tn*6814*, respectively. The results of all PCRs were positive, meaning that Tn*6765*, Tn*6813*, and Tn*6814* can be stably inherited in the bacteria.

## Discussion

The Tn*7*-like transposons are important mobile elements to transfer bacterial AMR. Compared with information obtained from studies about the transposition mechanism of the Tn*7*-like transposons, available data on the comprehensive analysis of Tn*7*-like transposons in *Enterobacterales* isolates are still inadequate. Herein, we determined the incidence of Tn*7*-like transposons in *Enterobacterales* isolates obtained from several farms of chicken and swine raised for meat purpose. Among the 1,474 *Enterobacterales* isolates, 377 strains that carry Tn*7*-like transposons were identified. In different genus of bacteria, the separation rate of Tn*7*-like transposons was different (*p* < 0.0001; Chi square test; Table 2). The positive rate of Tn*7*-like transposons in *Proteus* spp. (57.5%) was significantly higher than other bacteria’s (*p* < 0.0001; Chi square test). This finding was consistent with previous reports that genomes of *Proteus* spp. exhibited strong plasticity facilitating high-frequency insertion of mobile genetic elements like Tn*7*-like transposons (Dong et al., 2019; de Curraize et al., 2020; Gu et al., 2020). In addition, *E. coli* strains were ubiquitous commensal bacteria and abundant in the intestine of humans and animals (Zhang et al., 2013; Paitan, 2018). Therefore, its proportion in Tn*7*-like transposons positive *Enterobacterales* isolates was also large (Table 2). Since *E. coli*, *Proteus* spp. and some of other *Enterobacterales* species were opportunistic pathogens, even healthy food animals could be asymptomatic carriers of the bacteria and may cause an impact on human health. Our results provide evidence of the *Enterobacterales* strains as a possible reservoir of Tn*7*-like transposons, a risk that deserves our attention.

Antimicrobial susceptibility testing indicated that *Enterobacterales* strains carrying Tn*7*-like transposons exhibited high resistance to a variety of antibiotics, with a 54.9% multi-drug resistance rate. Relatively high rates of MDR in *Proteus* spp. (59.3%) and *S. enterica* (61.5%) strains may be due to the prevalence of multiple mobile elements in both bacteria, which was confirmed in previous studies (Beutlich et al., 2011; Murgia et al., 2015; Schultz et al., 2015; Lei et al., 2018). This also implied that Tn*7*-like transposons were likely to occur simultaneously with other mobile elements. High resistance to streptomycin (87.8%) and trimethoprim/sulfamethoxazole (74.0%) can be attributed to the *intI2*-associated resistance gene cassette (*aadA1*, *sat2*, and *dfrA1*) carried by Tn*7*-like transposons (Tietze and Brevet, 1991; Kaushik et al., 2019). The gene cassettes of *intI2* contained the aminoglycoside adenyltransferase (*aadA1* and *aadA2*), dihydrofolate reductase (*dfrA1*), and streptothricin acetyltransferase (*sat2*) encoding genes, which are responsible for streptomycin-spectinomycin, trimethoprim, and streptothricin resistance, respectively (Zhang et al., 2019). Through sequencing, we found that there were multiple mutated sites in *intI2*, among which 535 base mutations (C mutated to T) were terminating mutations (Figure 2). They led to premature termination of integrase gene translation, making its gene cassette sequence show a high degree of stability, usually *aadA1*, *sat2*, and *dfrA1* (Deng et al., 2015). Moreover, we knew from workers on the farms that spectinomycin is widely used against the respiratory or enteric infections in swine and chicken.

Compared with the resistance rates to streptomycin and trimethoprim/sulfamethoxazole, relatively low resistance rates to florfenicol (60.5%), gentamicin (24.4%), and ciprofloxacin (22%) were detected in the Tn*7*-like transposons positive strains. Most of them showed susceptibility to amikacin, ceftazidime, cefoxitin, aztreonam, and imipenem. The fact also emphasizes the importance of standardization of clinical drug application to prolong the efficacy of new drugs. The emergence of severe drug-resistant status in China is largely related to the abuse of antibiotics as feed additives in veterinary clinical practice and the transfer of movable components carrying drug-resistant genes between isolates, which has become a common problem in both human and veterinary clinical practice (Rahmani et al., 2013). Obviously, for *Enterobacterales*, carriage of Tn*7*-like transposons, each containing a set of resistance genes, may increase the chances of horizontal transfer of multiple resistance determinants to susceptible strains and may in turn bring unique advantages to the host and enable them to survive a strong antimicrobial selection pressure especially in poultry and livestock farm settings. Tn*7*-like transposons have been so successful at spreading into diverse relevant taxa that they could be used as a proxy for antibiotics pollution (Flores et al., 1992; Cleaver and Wickstrom, 2000).

In this study, six completed Tn*7*-like transposons genetic structures were identified by WGS analysis, which showed that they included three typical Tn*7* transposons (Tn*6763*, Tn*6764*, and Tn*6817*) and three novel Tn*7*-like transposons (Tn*6813*, Tn*6814*, and Tn*6765*). The IS*26* segment in Tn*6814* showed high level homology to the segment characterized in *E. coli* plasmid pV408 (GenBank accession number LN735560), and it harbored different mobile genetic elements (complete or truncated) and two resistance genes (*bla*_CTX-M-65_ and *fosA3*). The *bla*_CTX-M-65_-containing segment (IS*26*-*fipA*-IS*1380*-*bla*_CTX-M-65_-IS*903B*) in Tn*6814* also showed nucleotide identity to the corresponding genetic structure that habored *bla*_CTX-M-65_ gene in Tn*6813*, with the exception of one IS*26* sequence replaced by IS*903B* sequence in Tn*6814* (Figure 2). Interestingly, the organisms of Tn*6813* and Tn*6814* came from the same swine farm, and therefore, the *bla*_CTX-M-65_ segment bounded by two ISs in Tn*6814* could be derived from Tn*6813* or vice versa and likely by ISs-mediated mobilization.

Another resistance region bounded by IS*26* in Tn*6814* was identical to M1 module of pC271 in *E. coli* (GenBank accession number LN735561; Riccobono et al., 2015), containing resistance determinants to aminoglycosides [*aac(3)-IV* and *aph(4)-Ia*] and florfenicol (*floR*). Diverse mobile genetic elements in Tn*6814* included four intact ISs (three copies of IS*26*, an IS*903B*, and an IS*Ec9*) and one incomplete IS*1380*, while six intact ISs (four copies of IS*26*, an IS*6*, and an IS*Ec57*) and five incomplete elements (three IS*CR2*, an IS*1380*, and a Tn*5393*) were inserted in Tn*6813* (Figure 2). From the findings of various IS inserted sequences in these novel transposons, it is indicated that an increase in the diversity of Tn*7*-like transposons was prompted by the ISs-mediated homologous recombination.

Tn*6765* was highly homologous with Tn*6450* (GenBank accession number MF805806), which was located on the chromosome detected by Chen et al. (2018). The similarity of nucleic acid sequence was more than 99%, and the coverage was 96%. The differences between the two were mainly in the following four aspects: (i) Tn*6765* had a hypotheical protein gene before *bla*_DHA-1_; (ii) an *HypA* gene was inserted between *ampR* and *qacE1* in Tn*6765*; (iii) an incomplete aminoglycoside N(3)-acetyltransferase encoding gene was inserted between IS*Ec59* and *aph(4)-Ia*; and (iv) Tn*6450* was inserted downstream of the *glmS* gene, encoding glucosamine 6-phosphate synthase and surrounded by 5-bp direct repeats (CCAAT), whereas Tn*6765* was located on the plasmid, there is no *glms* gene and repeated sequences at both ends (Figures 3, 4). The carrier difference between Tn*6765* and Tn*6450* indicated that Tn*7*-like can be transmitted alternately on the chromosome and plasmid by cutting and inserting. To make matters worse, Tn*6450* comes from a chicken source (Chen et al., 2018) and Tn*6765* from a swine source suggested that Tn*7*-like transposons can be transmitted from one animal to another with bacterial hosts.

**Figure 5:**
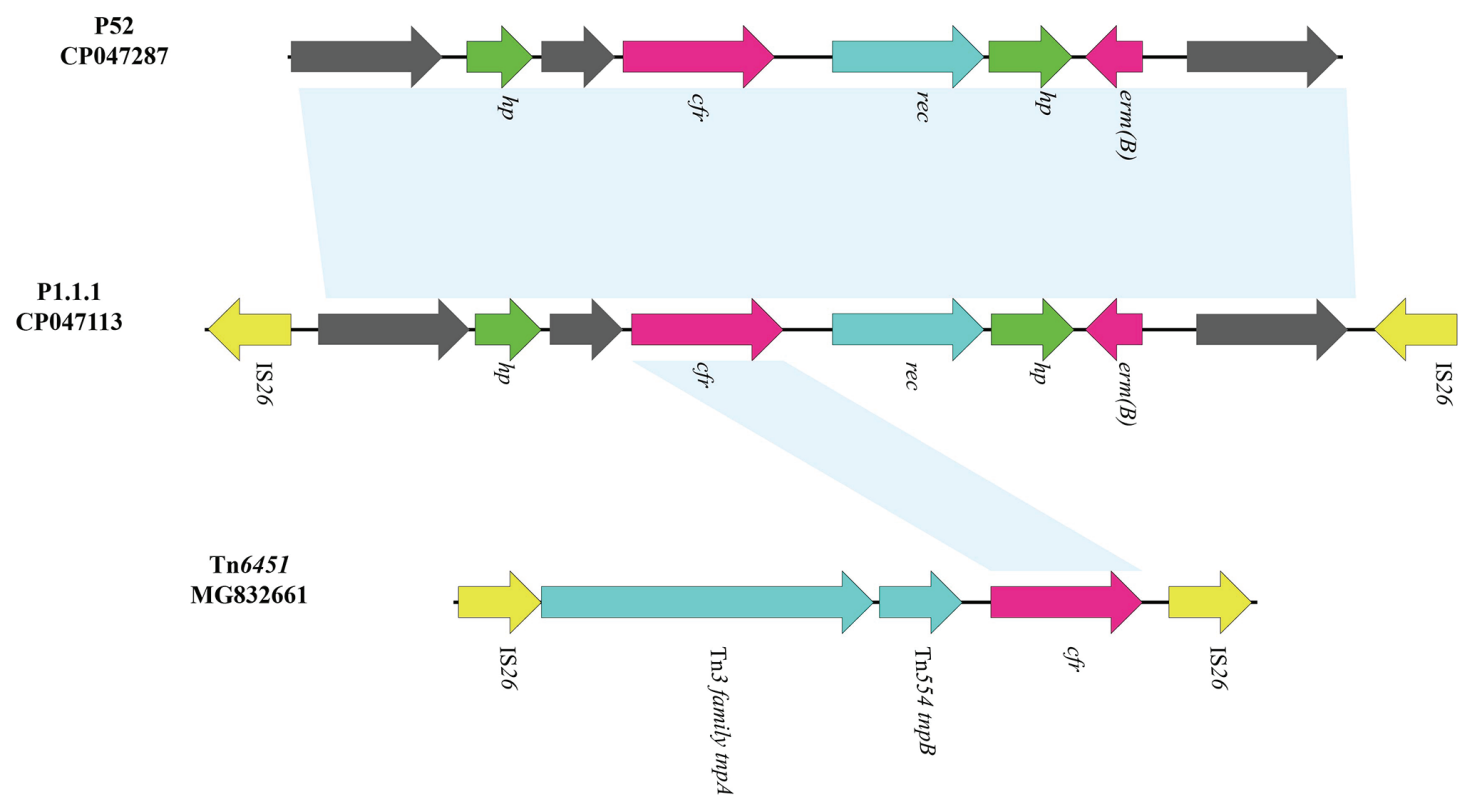
Genetic structure of *cfr*-containing region and in p1.1.1. The physical maps were generated using Easyfig 2.2.3 and DNAMAN Version 8.0. Linear comparison of the *cfr*-containing region in p1.1.1 with the *cfr*-containing region in *P. cibarius* strain G11 (Genebank number CP047287) and in *M. morganii* strain BCMM24 (Genebank number MG832661). Genes and ORFs are shown as arrows, and their orientations of transcription are indicated by the arrowheads. Antimicrobial resistance genes are in red. The IS elements are indicated by yellow arrows. Putative proteins are in green. Shared regions with 99% identity are indicated by shading.

The *cfr*-containing segment of plasmid p1.1.1 (corresponding to bases 8,925 to 12,726 to 4,624 in GenBank accession number CP047113) harbors a genetic structure, showing homology to the *cfr* segment characterized in *P. cibarius* G11 (Genebank accession number CP047287), which is partially differ from the existence of IS*26*-*cfr*-▵Tn*554 tnpB*-▵Tn*3* family *tnpA*-IS*26* section in another Tn*7*-like transposon, Tn*6451* (GenBank accession number MG832661; Chen et al., 2019; Figure 5). Although the *cfr* section of p1.1.1 and Tn*6451* is slightly different, this also suggests an evolutionary direction of the Tn*6765* carrying strain.

By conducting mating (conjugation) experiments, the Tn*6765*-carrying plasmid was successfully transferred to EC600. Although Tn*6813* and Tn*6814* located on the chromosomes could not be transferred by conjugation, the stability experiment showed that they could stably exist in the hosts. The “core transposition machinery” of Tn*7*-like transposon consists of the transposase proteins TnsA and TnsB along with a regulator protein, TnsC (Choi et al., 2013). This core machinery is directed by one of two target selecting proteins, TnsD or TnsE. Transposition with TnsABC+TnsD has evolved to maximize the efficiency of vertical transmission of the element by directing transposition into the chromosome. Transposition with TnsABC+TnsE occurs preferentially into mobile plasmids through the ability of the TnsE protein to recognize features found enriched during DNA replication on the lagging-strand template (Wolkow et al., 1996; Peters and Craig, 2000, 2001a). Therefore, it cannot rule out the possibility that these Tn*7*-like transposons located on the chromosome will target transposition into mobile plasmids, facilitating the spread of Tn*7*-like transposons between bacteria during the propagation of the hosts.

By comparing the genetic structure of different Tn*7*-like transposons, we can speculate that the multidrug-resistant Tn*7*-like transposons have a certain evolutionary relationship, which has contributed to Tn*7*-like transposons playing a vital role in the field of storing resistance genes. Although the assembly was original, each of these Tn*7*-like transposons, or parts thereof, was identical to those found in other plasmids or chromosomes. This was related to the fact that Tn*7*-like transposons can transfer between strains and accumulate genetic material *via* mobile genetic elements.

## Conclusion

This study is the first report to comprehensively analyze the incidence and antibiotic resistance characteristics of Tn*7*-like transposons in *Enterobacterales* isolates from livestock and poultry in China, and it presented detailed genetic characterization of three novel Tn*7*-like transposons Tn*6765*, Tn*6813*, and Tn*6814*, which were integrated into plasmids or chromosomes from *E. coli* and *P. mirabilis*. Multiple antibiotic resistance genes in Tn*7*-like transposons, considering that they were found in chicken and swine, are highly worrisome and may become a serious threat by spreading in other nearby animals, humans, and the environment. Therefore, robust measures should be taken to control the spread and emergence of mobile genetic resistance determinants in animals used for food production in China and the world, and our study provides an important reference for this.

## Data Availability Statement

The datasets presented in this study can be found in online repositories. The names of the repository/repositories and accession number(s) can be found in the article/Supplementary Material.

## Ethics Statement

The animal study was reviewed and approved by College of Life Science, Sichuan University affiliation ethics committee, and all efforts were made to minimize animal suffering.

## Author Contributions

All authors listed, have made substantial, direct and intellectual contribution to the work, and approved it for publication. JH conceived the study and drafted the manuscript. JH and CL collected samples and conducted data statistics. JH, CL, and PC performed experiments. HW supervised the research.

### Conflict of Interest

The authors declare that the research was conducted in the absence of any commercial or financial relationships that could be construed as a potential conflict of interest.

## Footnotes

^1^http://weblogo.berkeley.edu/logo.cgi

^2^http://blast.ncbi.nlm.nih.gov/Blast.cgi

^3^http://transposon.lstmed.ac.uk/
