# Supplementary material for: Detection of Tn7-Like Transposons and Antibiotic Resistance in Enterobacterales From Animals Used for Food Production With Identification of Three Novel Transposons Tn6813, Tn6814, and Tn6765
Source: Front Microbiol. 2020 Sep 4;11:2049. doi: 10.3389/fmicb.2020.02049 (PMC7500258; doi:10.3389/fmicb.2020.02049)
Supplement: Supplementary file 1 [file Data_Sheet_1.docx]

**Supplementary data**

Table S1. Primers used in this study.

| Location | Primer | Nucleotide sequence (5’-3’) | Product length (bp) | Reference |
| --- | --- | --- | --- | --- |
| *tnsA* | *tnsA-fw* | TGGCTAACAGTACAAGAAGT | 713 | This study |
| *tnsA* | *tnsA-rv* | CGCAACTCCTCCATATTCA |  |  |
| *tnsB* | *tnsB-fw* | GGCTGAGTTGTTGCTAATG | 845 | This study |
| *tnsB* | *tnsB-rv* | CCACCACATAAGACGGATT |  |  |
| *tnsC* | *tnsC-fw* | TCGCATAATGGTTCGCTAA | 893 | This study |
| *tnsC* | *tnsC-rv* | CTTGTCATCGTTGGATTCTG |  |  |
| *16s* | *16s-fw* | AGAGTTTGATCMTGGCTCAG | 1466 | ([Suzuki et al., 2000](#_ENREF_2)) |
| *16s* | *16s-rv* | TACGGYTACCTTGTTACGACTT |  |  |
| *intI2* | *intI2-fw* | CACGGATATGCGACAAAAAGGT | 788 | ([Mazel et al., 2000](#_ENREF_1)) |
| *intI2* | *intI2-rv* | GTAGCAAACGAGTGACGAAATG |  |  |

**References**

Mazel, D., Dychinco, B., Webb, V. A., & Davies, J. (2000). Antibiotic resistance in the ECOR collection: integrons and identification of a novel aad gene. *Antimicrob Agents Chemother, 44*(6), 1568-1574. doi: 10.1128/aac.44.6.1568-1574.2000

Suzuki, M. T., Taylor, L. T., & DeLong, E. F. (2000). Quantitative analysis of small-subunit rRNA genes in mixed microbial populations via 5'-nuclease assays. *Appl Environ Microbiol, 66*(11), 4605-4614. doi: 10.1128/aem.66.11.4605-4614.2000

Table S2. Antibiotic resistance rates of Tn*7*-like transposons positive strains isolated from different sources

| Antimicrobial  Agent | % resistant isolates from different sources | | P value |
| --- | --- | --- | --- |
|  | Pigs  (n=303) | Chicken  (n=74) |  |
| GEN (10μg) | 25.7 | 18.9 | 0.2205 |
| STR (10μg) | 88.8 | 83.8 | 0.2392 |
| FLR (30μg) | 60.7 | 59.5 | 0.7606 |
| CAZ (30μg) | 5.6 | 6.8 | 0.7061 |
| IPM (10μg) | 0.7 | 0.0 | 0.4835 |
| FOX (30μg) | 5.9 | 5.4 | 0.8602 |
| SXT(1.25/23.75μg) | 74.6 | 71.6 | 0.6020 |
| ATM (30μg) | 4.0 | 5.4 | 0.5804 |
| CIP (5μg) | 21.8 | 23.0 | 0.8246 |
| AMK (30μg) | 6.3 | 5.4 | 0.7804 |
| MDR | 55.4 | 52.7 | 0.6708 |

Chi-square test was used to analyze the data.

Table S3. Resistance Phenotype of EC600 with Plasmid p1.1.2

| Antibiotics | Inhibitory zone diameter（mm） | | | Antimicrobial resistance phenotype | | |
| --- | --- | --- | --- | --- | --- | --- |
|  | SCBX1.1 | EC600 | EC600+P1.1.2 | SCBX1.1 | EC600 | EC600+P1.1.2 |
| GEN(10μg) | 9 | 24 | 8 | R | S | R |
| STR (10μg) | 0 | 27 | 0 | R | S | R |
| FLR(30μg) | 0 | 24 | 0 | R | S | R |
| CAZ(30μg) | 13 | 30 | 11 | R | S | R |
| FOX(30μg) | 13 | 30 | 0 | R | S | R |
| SXT(1.25/23.75μg) | 0 | 31 | 0 | R | S | R |
| CIP(5μg) | 11 | 27 | 0 | R | S | R |
| AMK(30μg) | 0 | 23 | 0 | R | S | R |

R, resistant; I, intermediate; S, susceptible.


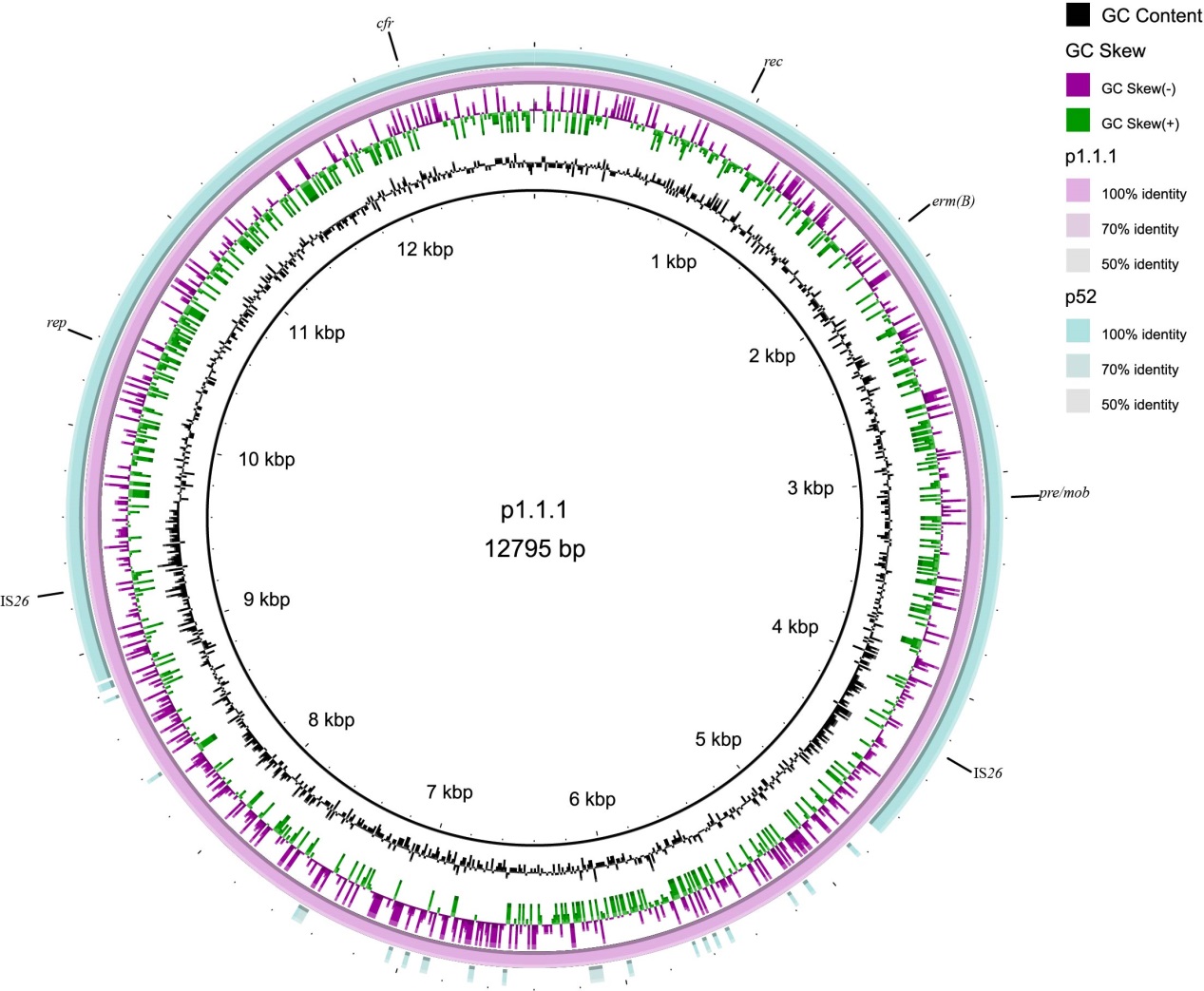


Fig. S1. Circular representation of plasmid p1.1.1. The physical map of p1.1.1 was generated using BRIG v0.95.
